# SDFormer: A Novel Transformer Neural Network for Structural Damage Identification by Segmenting the Strain Field Map

**DOI:** 10.3390/s22062358

**Published:** 2022-03-18

**Authors:** Zhaoyang Li, Ping Xu, Jie Xing, Chengxing Yang

**Affiliations:** 1 School of Traffic and Transportation Engineering, Central South University, Changsha 410075, China; zhaoyangli@csu.edu.cn (Z.L.); xuping@csu.edu.cn (P.X.); xingjie@csu.edu.cn (J.X.); 2 Key Laboratory for Track Traffic Safety of Ministry of Education, Central South University, Changsha 410075, China; 3 Joint International Research Laboratory of Key Technology for Rail Traffic Safety, Central South University, Changsha 410075, China

**Keywords:** structural damage identification, strain field, transformer neural network, deep learning, segmentation

## Abstract

Damage identification is a key problem in the field of structural health monitoring, which is of great significance to improve the reliability and safety of engineering structures. In the past, the structural strain damage identification method based on specific damage index needs the designer to have rich experience and background knowledge, and the designed damage index is hard to apply to different structures. In this paper, a U-shaped efficient structural strain damage identification network SDFormer (structural damage transformer) based on self-attention feature is proposed. SDFormer regards the problem of structural strain damage identification as an image segmentation problem, and introduces advanced image segmentation technology for structural damage identification. This network takes the strain field map of the structure as the input, and then outputs the predicted damage location and level. In the SDFormer, the low-level and high-level features are smoothly fused by skip connection, and the self-attention module is used to obtain damage feature information, to effectively improve the performance of the model. SDFormer can directly construct the mapping between strain field map and damage distribution without complex damage index design. While ensuring the accuracy, it improves the identification efficiency. The effectiveness and accuracy of the model are verified by numerical experiments, and the performance of an advanced convolutional neural network is compared. The results show that SDFormer has better performance than the advanced convolutional neural network. Further, an anti-noise experiment is designed to verify the anti-noise and robustness of the model. The anti-noise performance of SDFormer is better than that of the comparison model in the anti-noise experimental results, which proves that the model has good anti-noise and robustness.

## 1. Introduction

The long-term use of engineering structures often results in structural aging and fatigue due to long-term external force load and the influence of various physical and chemical factors, resulting in changes in size, shape, and their own material properties. The resulting structural damage threatens the safe use of engineering structures. Therefore, structural damage identification has always been a key issue in the field of structural health monitoring. For large engineering structures, especially the key components that were hard to disassemble for separate maintenance, non-destructive testing (NDT) technology was mainly used to carry out regular maintenance and testing on engineering structural parts. Its technical means include magnetic particle flaw [1], ultrasonic [2], X-ray [3], eddy current [4], etc. However, these methods had some limitations. Magnetic particle testing and eddy current testing were affected by the material and structure of the tested parts, and it was hard to detect the deep internal cracks in the structure. Ultrasonic testing was easy to operate and has realized automatic testing, but it was not suitable for the identification of complex structures, and it could not achieve real-time identification because of its low efficiency. X-ray testing had a high detection efficiency, but it had radiation impact on long-term workers. Moreover, these methods require inspectors to have rich experience and there is a need to confirm and process the test data manually. These shortcomings virtually increase the test cost.

With the progress of detection sensor technology, especially the emergence of fiber Bragg grating sensing technology [5] and digital speckle measurement technology [6], the acquisition of strain field data becomes convenient, and the damage identification method based on strain modal data is widely used because of the high sensitivity of strain to local damage. In the field of structural damage identification based on strain mode information, the traditional method was to design a sensitive and accurate damage index to identify the damage in the tested structure. Kaewunruen et al. [7] proposed a damage identification index based on curvature analysis based on the strain measured by the fiber Bragg grating strain sensor, which was used to identify the damaged cavity under railway sleeper. WL Bayissa et al. [8] proposed spectral or mean strain energy (SSE) for structural damage identification of plate-like structures, and a large number of numerical simulation studies show that SSE index had high sensitivity to damage and good identification performance. Fariba et al. [9] proposed a strain frequency response function (SFRF) for strain damage identification of structures, and studies showed that their method had low error rate and strong anti-noise ability.

However, the method of designing strain damage index needs a deep understanding and background knowledge of the detected structure, and it was hard to migrate to different application scenarios and different structures. Due to the popularity of sensors, structural health monitoring has higher and higher requirements for real-time monitoring, and a large number of sensor data need to be processed. The method based on strain damage index struggled to overcome the problem of low data processing efficiency caused by data explosion, and the environmental noise and unpredictable load in the actual scene would lead to large deviation of strain damage index.

In recent years, with the rapid development of deep learning technology and big data technology, the data-driven structural health monitoring had become a research hotspot. Osama Abelijaber et al. [10] achieved damage location of truss steel structure by using a one-dimensional convolutional neural network. Seokgoo Kim [11] proposed a convolutional neural network based on signal segmentation to identify the damage of different gears and achieved good results. Bao [12] imagized one-dimensional time series data and trained the deep neural network on this basis, which was applied to abnormal acceleration data detection of a long-span cable-stayed bridge, and achieved an accuracy of 87% with satisfactory results. Hyo et al. [13] used convolutional neural network to predict the strain changes of key structural points of concrete, to evaluate the life of concrete structures. Jinhua Lin et al. [14] used X-ray detection images of forged parts to train convolutional neural network for damage identification of forged parts, and obtained an accuracy rate of more than 96% with high identification efficiency. Zhang et al. [15] proposed an improved deep learning algorithm for crack identification on asphalt pavement. Their improvement gave the algorithm higher accuracy and significantly reduced the probability of misjudgment. Kumar et al. [16] proposed a two-stage fault detection and classification method designed for a rotating motor, which combines numerical features and shallow neural network. Jin and Chen [17] proposed a novel end-to-end intelligent vibration signal classification framework based on modified transformer neural network, and proved that the proposed method is better than the signal classification method based on convolution neural network and recurrent neural network through two cases.

The structural damage location and identification method based on deep learning could adaptively extract the structural feature information from the original response data for damage identification. However, the current research is still limited to images or strain mode data of several key nodes for structural damage identification. Those methods were restricted by external conditions such as insufficient illumination and detection points, which limits its application scope. There was still a lack of a mapping method between strain field data and structural damage information to realize real-time damage identification. In recent years, the rapid development of deep learning in the field of image semantic segmentation provides a new way to realize this idea. Fully convolutional network (FCN) [18] was the first end-to-end deep convolution neural network (CNN) for image semantic segmentation. It brings image semantic segmentation into the era of deep learning. After FCN, the DeepLab [19] series and the PSPnet [20] network had brought far-reaching hole convolution and feature pyramid structure, respectively, and their design ideas have influenced others so far. UNet is the first deep convolution neural network using symmetrical encoder-decoder structure for medical image segmentation. After combining with residual network (ResNet) [21], the effect has been greatly improved, and its simple symmetrical structure has been borrowed by most later deep neural networks. Since 2021, the vision transformer network, represented by ViT [22], has defeated the traditional CNN network in many tasks. In particular, the emergence of the swin transformer [23] has brought the whole field of image segmentation into a new era. The accuracy of image semantic segmentation is improving with the development of deep learning. At present, there is still no relevant research trying to use image segmentation technology to segment the strain field for structural damage identification. However, the research on damage assessment of composite laminated structures by Tran-Ngoc et al. [24] and Samir Khatir et al. [25] gives enlightenment to this study. In their research, the composite structure to be detected is first meshed, and then damage identification is carried out based on specific damage indicators combined with artificial neural network. Their research is still limited to designing specific damage indicators for damage identification, but the idea of meshing the detection area and then identifying each grid is worth learning from. Therefore, when the mesh of the structure is dense enough, the problem of structural damage identification can be regarded as an image segmentation problem, and then the current advanced depth image segmentation algorithm can be used for structural damage identification.

In this paper, the structural damage identification problem based on strain field data is regarded as an image semantic segmentation problem. With the help of deep learning technology, a structural strain damage identification network combining encoder–decoder configuration and advanced transformer structure, SDFormer, is proposed. This neural network was trained by the simulation results, and the predicted damage map of the structure was predicted from the strain field map. Compared with the real damage map, the feasibility of the model was verified.

To present our method, this paper is organized as follows: Section 2 introduces the process of the damage identification method and the proposed network architecture, Section 3 describes the setup of the numerical experiment and results of the method, Section 4 discusses the advantages and disadvantages of the method, and Section 5 contains the conclusion of this paper.

## 2. Proposed Method

### 2.1. Overall Architecture

**Figure 1:**
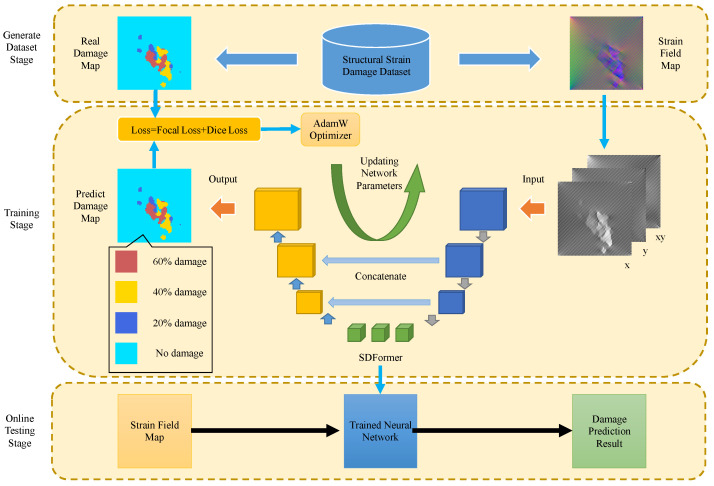
The structural strain damage identification framework.

In this paper, a U-shaped network, structural damage transformer (SDFormer), which is based on swin transformer [23], is proposed for structural strain damage identification. The network takes the strain field as input and outputs the corresponding damage position and level. The structural strain damage identification framework in this paper includes three stages: generate strain damage dataset stage, training stage, and online testing stage, as shown in Figure 1.

In the generate dataset stage, there are two ways to build the dataset: using the strain field map detected by sensors or using the strain field map simulated by finite element model. The dataset should include two parts: 1. strain field map; 2. the real damage map, which should correspond to the strain field map one-to-one. The cost of tagging the strain field map dataset detected by sensors is prohibitive. Therefore, in this paper, the strain field map simulated by finite element model is used to build the dataset. We preset the structural damage location and level, and built the corresponding finite element model, then obtained the simulated strain field map as the input of the neural network, and the preset structural damage map as the real damage map label of the neural network. The setting method of simulated damage is introduced in Section 3.

In the training stage, the strain field data are processed into tensors with the size of H×W×N, where *H* and *W* represent the length and width of the strain field, and *N* is the number of channels of the strain field data. It corresponds to the strain data in *x*, *y*, and xy directions respectively, N=3. Then, the processed strain field data is used as the input of the SDFormer. The SDFormer outputs the prediction probability tensor *P* with the size of H×W×Cls, where *H* and *W* correspond to the size of the strain field, Cls is the number of predicted damage categories, Cls=d+1, where *d* is the number of preset strain damage levels, and 1 refers to the normal category without damage. Three strain damage levels of 20%, 40%, and 60% are preset in this paper. Therefore, Cls set in this paper is 4. $P_{i,j,k}$ is the probability of *k*-th damage of the element with coordinates (i,j). When k=0, it represents the probability of no strain damage of the element. The real damage map *T* is processed into a [0,1] tensor with the size of H×W×Cls, too. $T_{i,j,k}=1$ means that the element with coordinate (i,j) produces the *k*-th damage. Then, *P* and *T* will be used to calculate the gradient of the loss function to the SDFormer parameters, and use the AdamW [26] algorithm to update the SDFormer parameters. This process is cycled until loss converges to obtain the trained SDFormer.

In the online testing stage, the real-time detected strain field data is processed into the format which is required by the network and input into SDFormer, then the output of SDFormer is the predicted damage map.

### 2.2. SDFormer

**Figure 2:**
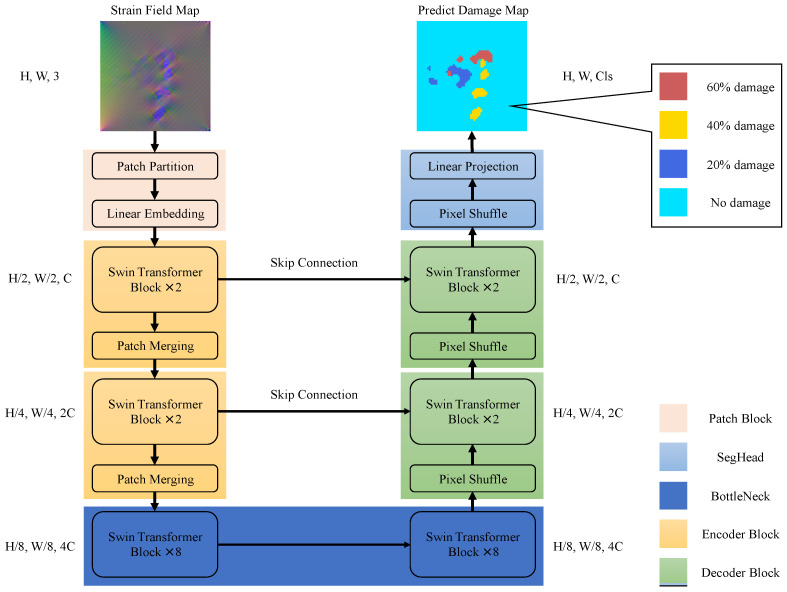
Overview of SDFormer network architecture.

The architecture of the proposed SDFormer in this paper is presented in Figure 2. The SDFormer consists of patch block, encoder block, bottleneck, decoder block, and seghead, in which the symmetrical encoder and decoder blocks are connected by skip connection.

In the patch block, the strain field map *S* would be split into several non-overlapping patches with a size of 2×2, and then flattened. These flattened patches would be transformed into vectors with length *C* by an linear embedding layer. The encoder block consists of two swin transformer blocks and a patch merging layer. The feature map from the previous layer would be processed by the swin transformer block first, and the processed feature map is retained. The processed feature map would be sent to the patch merging layer and the decoder block symmetrical to the current encoder block at the same time. Then, patch merging downsamples the feature map to obtain the global feature information and send it to the next layer. This design is very common in the field of computer vision. In convolutional neural networks, a similar operation is the pooling layer. In fact, patch merging is similar to patch block, except that it further merges the split patches, so we call it patch merging. The bottleneck consists of two groups of symmetrical swin transformer blocks. We could control the complexity of the model to meet the actual needs by adjusting the number of swin transformer blocks in bottleneck. The decoder block performs the opposite operation to the encoder block. The input feature map first goes through a pixel shuffle [27] layer for upsampling, and then it would be concatenated with the feature map from the symmetrical encoder block in the channel dimension and sent to the swin transformer blocks, and the processed feature map would be sent to the next layer.

Finally, in SegHead, a pixel shuffle layer restores the feature map to the same size as the input strain field map, and then obtains the final damage prediction probability tensor *P* through the linear projection layer, which is a fully connected layer.

#### 2.2.1. Patch Block and Patch Merging

**Figure 3:**
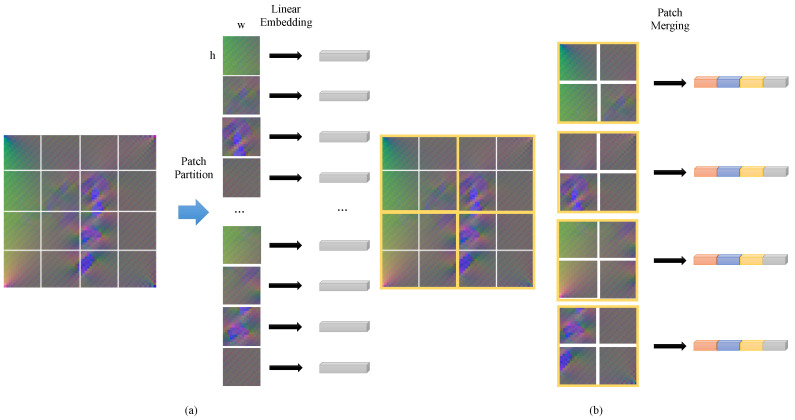
Patch block and patch merging: (**a**) Patch block: splits the strain field map and embeds them. (**b**) Patch merging: concatenates the patch and re-embeds.

In the patch block, the strain field map S is split into non-overlapping patches with a size of 2×2, and then flattened, as shown in Figure 3a. In this way, the size of S will change from H×W×3 to $\frac{H}{2}\times \frac{W}{2}\times 12$. Then, a fully connected layer is used to embed each patch, and the feature dimension of each patch is transformed into *C*. In this paper, C=64. The size of the output feature map F is $\frac{H}{2}\times \frac{W}{2}\times C$.

Patch merging merges the patches of the previous layer feature map, as shown in Figure 3b. Similar to patch block, patch merging uses a 2×2 sliding window to split the feature map and merge the patches in the window, so that its size changes from H×W×C to $\frac{H}{2}\times \frac{W}{2}\times 4C$. Then, a fully connected layer is used to reduce the dimension of the embedded feature from 4C to 2C. Thus, the size of the feature map output by patch merging is $\frac{H}{2}\times \frac{W}{2}\times 2C$. The calculation formula of patch merging is shown in Formulas (1) and (2).

(1) $$x^{l}=MLP\left(PatchMerging\left(x^{l-1}\right)\right)$$

(2) MLP(x)=wx+b

where $w\in R^{4C\times 2C}$ is the weight of the full connection layer and $b\in R^{2C}$ is the bias of the full connection layer. It is worth noting that $w\in R^{4C\times 2C}$ only affects the embedded feature dimension.

#### 2.2.2. Pixel Shuffle

Pixel shuffle [27] is a parameterless upper sampling layer. Its principle is to reorder an input tensor to complete the upper sampling of the input tensor. Its mathematical expression can be written as Formula (3)

(3) $$PixelShuffle{\left(T\right)}_{x,y,c}=T_{\lfloor x/r\rfloor ,\lfloor y/r\rfloor ,C\cdot mod(y,r)+C\cdot mod(x,r)+c}$$

where $T\in R^{H\times W\times C\cdot r^{2}}$ is the input tensor, *r* is the magnification of upsampling, and *C* is the number of channels of the output tensor. When the size of input tensor is $H\times W\times C\cdot r^{2}$, the size of output tensor is r·H×r·W×C.

In addition, a fully connected layer is added in front of the pixel shuffle layer to adjust the embedded feature dimension of the input feature map to meet the requirements of the pixel shuffle layer. In the SDFormer, all feature maps passing through the pixel shuffle layer will be upsampled to twice the original size.

#### 2.2.3. Swin Transformer Block

**Figure 4:**
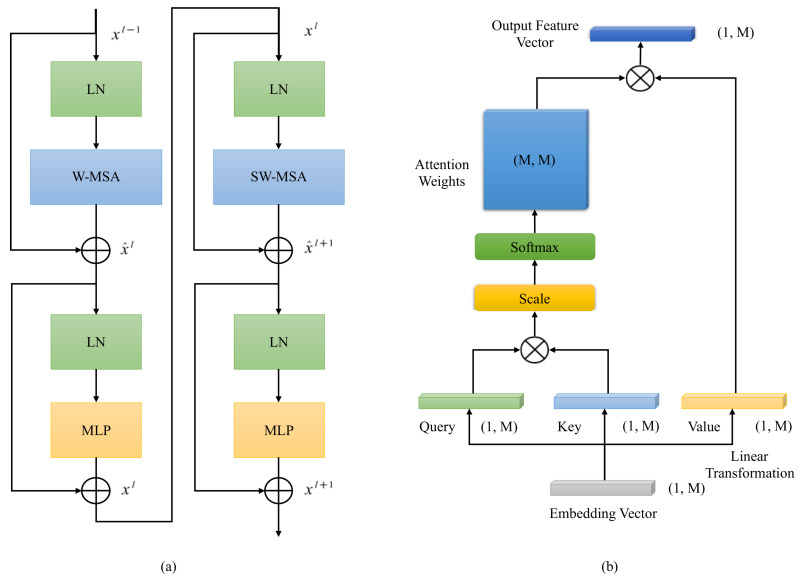
Overview of swin transformer block and the multi-head self-attention (MSA) module. (**a**) Architecture of swin transformer block. (**b**) Calculation flow of multi-head self-attention (MSA) module.

The basic unit of SDFormer is the swin transformer block, which is an important part in the encoder block, decoder block, or bottleneck. Different to the traditional multi-head self-attention module, the swin transformer block is constructed by the multi-head attention module based on fixed window and shifted window. The structure of the swin transformer block is shown in Figure 4a, which includes four layernorm (LN) layers, a window-based multi-head self-attention module (W-MSA), a shifted window-based multi-head self-attention module (SW-MSA), and two multilayer perceptron (MLP) layers with nonlinear activation function GeLU.

W-MSA splits the feature map into several non-overlapping windows, and then calculates the local self-attention in each window, which can reduce the computational complexity significantly while maintaining the global attention. At the same time, SW-MSA is proposed to solve the disadvantage of lack of cross-window connection in W-MSA and enhance the capability of the whole model, and cross-window connection is introduced on the basis of SW-MSA and maintains its efficient computing power. The difference between SW-MSA and W-MSA is that a window shift operation is added before W-MSA. This small change makes the whole network model better able to deal with the global information. In practical implementation, shifting the feature map achieves the same effect. W-MSA and SW-MSA are used alternately to form a swin transformer block, which can be expressed by the following formula:

(4) $${\hat{x}}^{l}=W\text{-}\mathrm{MSA}\left(LN\left(x^{l-1}\right)\right)+x^{l-1}$$

(5) $$x^{l}=MLP\left(LN\left({\hat{x}}^{l}\right)\right)+{\hat{x}}^{l}$$

(6) $${\hat{x}}^{l+1}=\mathrm{SW}\text{-}\mathrm{MSA}\left(LN\left(x^{l}\right)\right)+x^{l}$$

(7) $$x^{l+1}=MLP\left(LN\left({\hat{x}}^{l+1}\right)\right)+{\hat{x}}^{l+1}$$

(8) $$LN\left(x\right)=\frac{x-E[x]}{\sqrt{Var(x)+\epsilon }}\cdot \gamma +\beta$$

where ${\hat{x}}^{l}$ and $x^{l}$ are the feature maps output by the ith (S)W-MSA module and MLP module, respectively, E[x] is the mean value of *x*, Var(x) is the variance of *x*, ϵ is usually $1\times 10^{-5}$, and γ and β are learnable affine transformation parameters. When calculating self-attention, similar to previous studies [28,29], we first use an MLP layer to map the input embedding feature vector into three vectors of the same size: query, key, and value. Then we calculate the similarity between query and key, obtain an attention weight matrix after passing through a softmax layer, and then multiply the attention weight matrix and the value to obtain the final output. The formula of self-attention is as follows:

(9) $$Attention\left(Q,K,V\right)=SoftMax\left(\frac{QK^{T}}{\sqrt{d}}+B\right)V$$

where $Q,K,V\in R^{M^{2}\times d}$ are query, key, and value respectively, *M* is the window size for calculating local self-attention, *d* is the dimension size of query or key, and $B\in R^{M^{2}\times M^{2}}$ represents an offset matrix. Since storing an $M^{2}\times M^{2}$ matrix requires a lot of memory, and the relative position of the patch in the window is often between [−M+1,M−1], a $\hat{B}\in R^{\left(2M-1)\times (2M-1\right)}$ is usually used to save the value of *B*.

### 2.3. Loss and Optimizer

Subtle strain damage is very common in engineering structures. This makes the area of strain damage account for a small part in the strain field map. Then, there is a serious sample imbalance in the training data, which makes the model hard to fit. In order to solve this problem, the following loss function is designed for model training:

(10) $$L\left(y,p\right)=L_{dice}\left(y,p\right)+L_{focal}\left(y,p\right)$$

(11) $$L_{dice}\left(y,p\right)=1-\frac{2|y\cap p|}{\left|y|+|p\right|}$$

(12) $$L_{focal}\left(y,p\right)=-\alpha y{\left(1-p\right)}^{\gamma }log\left(p\right)-\left(1-\alpha \right)\left(1-y\right)p^{\gamma }log\left(1-p\right)$$

where *y* is the real damage map, *p* is the predicted damage map, |y∩p| is the area of the intersection area between the real damage map and the predicted damage map, |y| is the area of the real damage map, |p| is the area of the predicted damage map, α and γ are adjustable super parameters, α defaults to 0.5, and γ defaults to 2. It can be seen that when the predicted damage distribution is more similar to the real damage distribution, the dice loss ($L_{dice}$) [30] is smaller. However, when dice loss encounters small damage that is difficult to distinguish, there will be a problem that the overall loss value is very small but the prediction effect is poor, resulting in difficulty in model convergence. Therefore, we add a focal loss ($L_{focal}$) [31] item to increase the loss of these difficult samples, making the network more sensitive to such damage. In the training process, AdamW [26] algorithm was used to train the model. AdamW algorithm is an improved version of Adam [32], and its mathematical formula is as follows:

(13) $$g_{t}=\nabla f_{t}\left(\theta_{t-1}\right)$$

(14) $$m_{t}=\beta_{1}m_{t-1}+\left(1-\beta_{1}\right)g_{t}$$

(15) $$v_{t}=\beta_{2}v_{t-1}+\left(1-\beta_{2}\right)g_{t}^{2}$$

(16) $${\hat{m}}_{t}=m_{t}/\left(1-\beta_{1}^{t}\right)$$

(17) $${\hat{v}}_{t}=v_{t}/\left(1-\beta_{2}^{t}\right)$$

(18) $$\theta_{t}=\theta_{t-1}-\eta_{t}\left(\alpha {\hat{m}}_{t}/\left(\sqrt{{\hat{v}}_{t}}+\epsilon \right)+\lambda \theta_{t-1}\right)$$

where $\theta_{t}$ is the network model parameter at time *t*; $g_{t}$ is the gradient of model parameters at time t−1; $\eta_{t}$ is the learning rate at time *t*; $m_{t}$ is the first moment vector, $m_{t=0}=0$; $v_{t}$ is the second moment vector, $v_{t=0}=0$; $\hat{m_{t}}$ is the first moment vector after correcting the offset; $\hat{v_{t}}$ is the second moment vector after correcting the offset; $\beta_{1}$ and $\beta_{2}$ default to 0.9 and 0.999 respectively; α defaults to 0.001; ϵ defaults to $1\times 10^{-8}$; λ is the weight attenuation coefficient, and it defaults to 0.01.

## 3. Numerical Experiments and Results

In this section, two finite element simulation datasets were constructed to verify the effectiveness of the proposed SDFormer. Moreover, this study compares SDFormer with three popular CNN methods, including UNet [33], PSPnet [20], and DeepLabV3 [19], where ResNet-50 [21] was taken as the backbone network, to evaluate the performance of the SDFormer. The loss function and optimization algorithm used in these network training are consistent with SDFormer, as mentioned in Section 2.3.

### 3.1. Numerical Experiments Setup

#### 3.1.1. Damage Simulation

It is a simple and practical method to simulate material damage by replacing the elastic modulus of the original material with that of the damaged material. This method, based on strain equivalence hypothesis, has been widely used because of its simple implementation. In the strain equivalence hypothesis, the deformation of damaged material due to stress is equivalent to the deformation of a hypothetical non-destructive material under stress, and the actual effective bearing area of damaged material is equal to the virtual bearing area of non-destructive material. Its mathematical formula is as follows:

(19) $$\frac{\sigma^{\prime }}{E}=\frac{\sigma }{E^{\prime }}$$

(20) $$\sigma^{\prime }=\frac{\sigma }{1-D}$$

where *E* is the elastic modulus of undamaged material, $E^{\prime }$ is the elastic modulus of damaged material, σ is the nominal stress, and $\sigma^{\prime }$ is the effective stress; *D* is the damage factor, and its value is between 0 and 1. Combining Formulas (19) and (20):

(21) $$D=1-\frac{E^{\prime }}{E}$$

It can be seen that when there is no damage, D=0, and the elastic modulus of the material does not change. When complete damage occurs, D=1, and the elastic modulus of the material becomes 0. Based on Formula (21), this study sets up three kinds of damage in the case of D=0.2, D=0.4, and D=0.6 to construct the datasets.

In Case 1 and Case 2, the damage simulation is carried out according to the following steps:

1. For the structural component *S*, the material Mat of *S* and the corresponding elastic modulus *E* are known. The preset damage level D∈(0.2,0.4,0.6) defines four materials (three damaged materials and one undamaged material), in which the undamaged material corresponds to Mat, the elastic modulus of the three damaged materials is set as $E^{\prime }=E\left(1-D\right)$, according to Formula (21), and other material properties are consistent with Mat.
2. Mesh *S*, and randomly select an area not exceeding 5% of the total area of the structure for each damaged material. If the selected area of two damaged materials overlaps, the one set later is the material of the overlapping area. In addition, other conditions are consistent, and a damage sample finite element model is obtained.
3. The damage map and corresponding strain field data in X, Y, and XY directions are obtained by solving the finite element model obtained in step 2.
4. Repeat steps 2 and 3 until enough data samples are obtained to construct the structural strain damage dataset.

#### 3.1.2. Case 1

**Figure 5:**
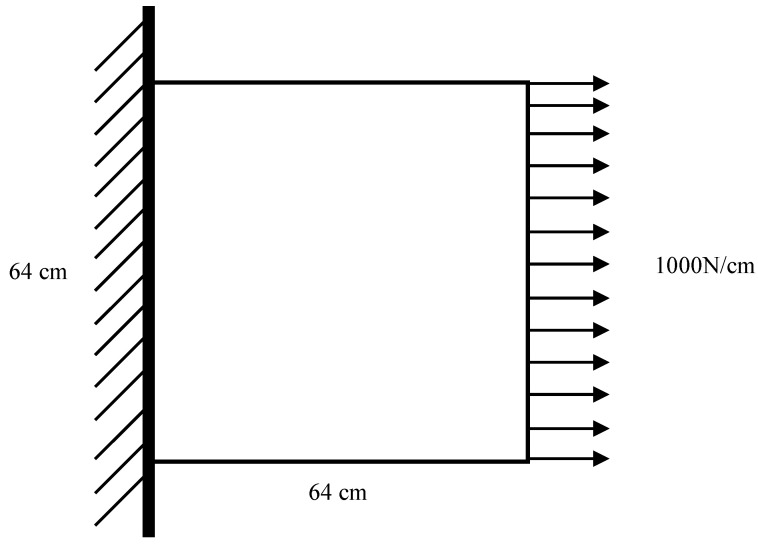
Schematic diagram of plate finite element model.

In this case, a simply supported plate model was constructed, as shown in Figure 5. The simply supported plate is a flat plate with a size of 640 mm × 640 mm composed of shell elements. One end of the plate was fixed, and the other end was applied with a uniformly distributed load of 1000 N/cm in the positive direction of the *X*-axis.

The default material of the plate is aluminum, the elastic modulus is 70 Gpa, the Poisson’s ratio is 0.33, and the density is 2700 kg/m^3^. In this case, three damage levels were set as mentioned in Section 3.1.1, i.e., 20%, 40%, and 60% material damage. Three hypothetical materials were defined to simulate this damage. The elastic moduli of these three hypothetical materials were 80%, 60%, and 40% of aluminum, respectively. Other material properties are consistent with aluminum.

**Figure 6:**
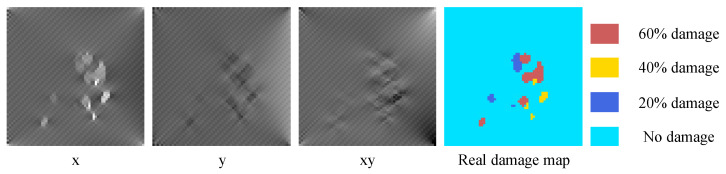
Example of plate dataset.

Following the four steps of damage simulation in Section 3.1.1, the plate model was meshed according to the size of 10 mm × 10 mm, and 4096 finite elements were obtained. The strain field data obtained by solving the finite element model would be saved as a tensor with the size of 64 × 64 × 3 as the strain field map in the dataset, and the damage selection area would be saved as the real damage map $T^{\left(64\times 64\right)}=\left(t_{ij}\right)$, where $t_{ij}\in \left(0,1,2,3\right)$ refers to the damage level D=(0,0.2,0.4,0.6). This process is repeated to obtain 2000 groups of data samples under different damage conditions. In this paper, a Python script was used to call ABAQUS to build this dataset. And an example of the plate dataset is shown in Figure 6.

#### 3.1.3. Case 2

**Figure 7:**
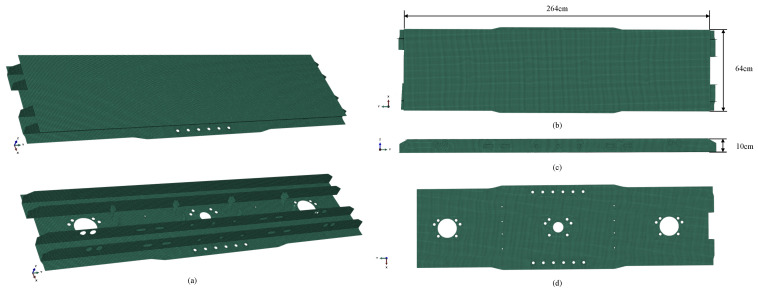
Schematic diagram of sleeper beam. (**a**) Overall structure of the sleeper beam. (**b**) Top view of the sleeper beam, the length of the cover plate is 264 cm and the width is 64cm. (**c**) Front view of the sleeper beam, the height of the sleeper beam is 10 cm. (**d**) The lower cover plate of the sleeper beam.

In this case, a strain damage dataset of a sleeper beam of a metro train is constructed. The sleeper beam of the metro train is shown in Figure 7a, and is composed of a cover plate, a bottom plate, and reinforcing structures in the middle.

This case takes the strain damage identification of the cover plate of a sleeper beam as the research task, as shown in Figure 7b. The cover plate of the sleeper beam is a rectangular non-perforated plate. As an important component of bearing and connecting the vehicle body and traveling part, the state of the cover plate is related to the driving safety of the train. However, the damage of the sleeper cover plate is hard to detect because of the particularity of the sleeper position. Thus, this case expects to use the method proposed in this paper to conduct the real-time strain damage identification of the sleeper beam cover plate.

In this case, the size of the cover plate of the sleeper beam was 2640 mm × 640 mm × 10 mm. The bottom plate of the sleeper beam is fixed, and a uniform load of 0.3 Mpa in the −z direction is applied to the cover plate. The default material of the sleeper beam is steel, the elastic modulus is 206 GPa, the Poisson’s ratio is 0.3, and the density is 7850 kg/m${}^{3}$. Similar to Case 1, this case also sets the same three damage levels. Three hypothetical materials were defined to simulate this damage. The elastic moduli of the three hypothetical materials are 80%, 60%, and 40% of steel, respectively. Other material properties were consistent with steel.

**Figure 8:**
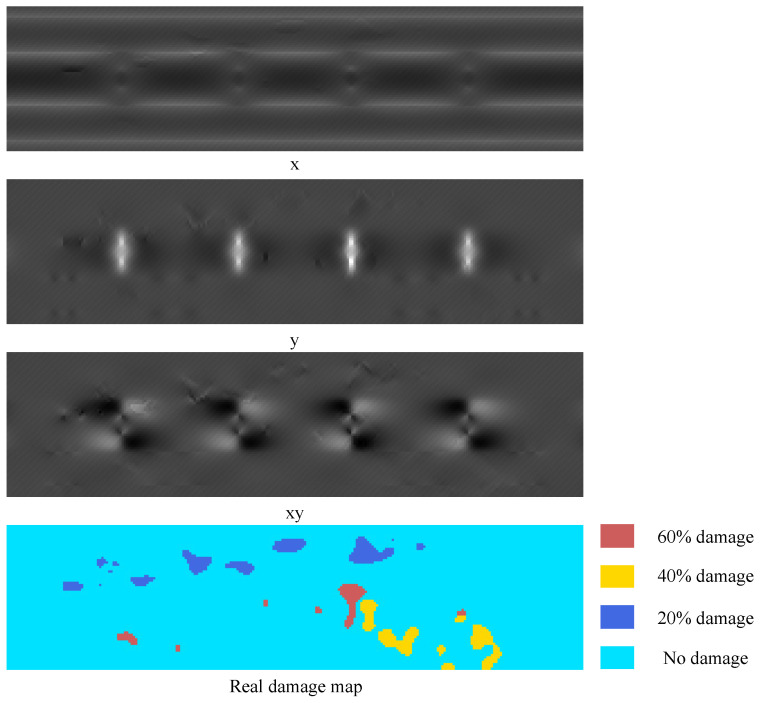
Example of sleeper beam dataset.

Following the four steps of damage simulation in Section 3.1.1, the cover plate was meshed according to the size of 10 mm × 10 mm, and 16,896 finite elements were obtained. The strain field data obtained by solving the finite element model would be saved as a tensor with the size of 264×64×3 as the strain field map in the dataset, and the damage selection area would be saved as the real damage map $T^{\left(264\times 64\right)}=\left(t_{ij}\right)$, where $t_{ij}\in \left(0,1,2,3\right)$ refers to the damage level D=(0,0.2,0.4,0.6). This process is repeated to obtain 2000 groups of data samples under different damage conditions. The dataset of this case is also built by calling ABAQUS with Python script. And an example of the sleeper beam dataset is shown in Figure 8.

#### 3.1.4. Evaluation Metrics

In this paper, mean intersection over union (MIoU) is used to evaluate the performance of each model. MIoU is calculated according to the following formula:

(22) $$MIoU=\frac{1}{k+1}\sum_{i=0}^{k}\frac{p_{ii}}{\sum_{j=0}^{k}p_{ij}+\sum_{j=0}^{k}p_{ji}-p_{ii}}$$

where $p_{ij}$ is the number of class *i* damage units predicted as class *j* damage in the network output results, and *k* is the number of damage categories.

### 3.2. Result

In this section, the datasets of Case 1 and Case 2 were divided into training set and testing set according to the ratio of 8:2, respectively, to test the performance of PSPnet [20], DeepLabV3 [19], UNet [33], and SDFormer. The maximum training epochs of all models including comparison models and the proposed model are 50; the batch size is 4; the initial learning rate is set to $10^{-5}$; and AdamW algorithm is selected as the model optimization algorithm.

**Figure 9:**
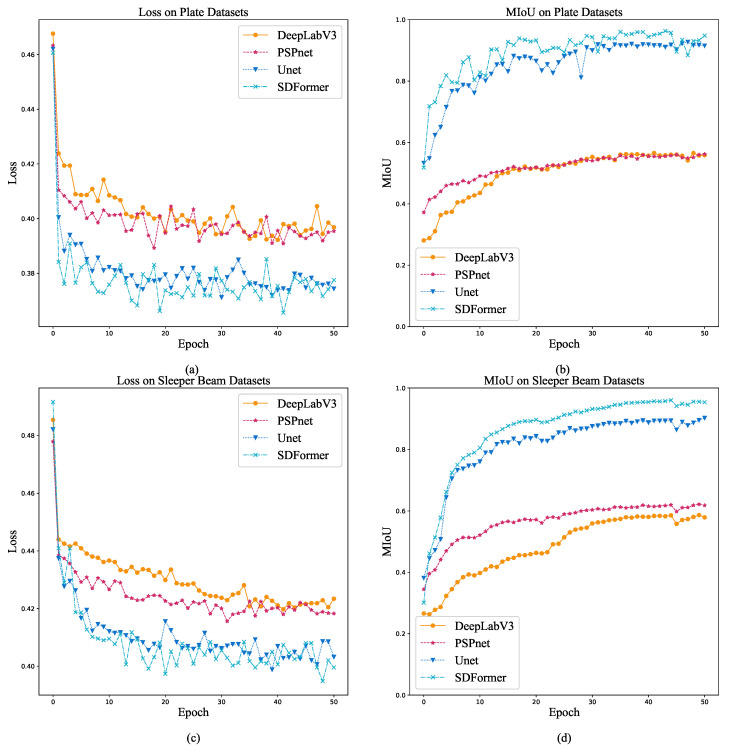
Training loss curve and MIoU curve. (**a**) Training loss curve on plate dataset. (**b**) Training MIoU curve on plate dataset. (**c**) Training loss curve on sleeper beam dataset. (**d**) Training MIoU curve on sleeper beam dataset.

Figure 9 shows the training loss curve and MIoU curve of DeepLabV3, PSPnet, UNet, and SDFormer on the plate dataset and the sleeper beam dataset. It could be found that under the same training conditions, SDFormer can make the loss function converge faster and obtain better MIoU performance than the other three models. It means that SDFormer has stronger capacity to extract features from the strain field map and a higher performance upper limit. This is very important for the accurate detection of structural damage.

**Figure 10:**
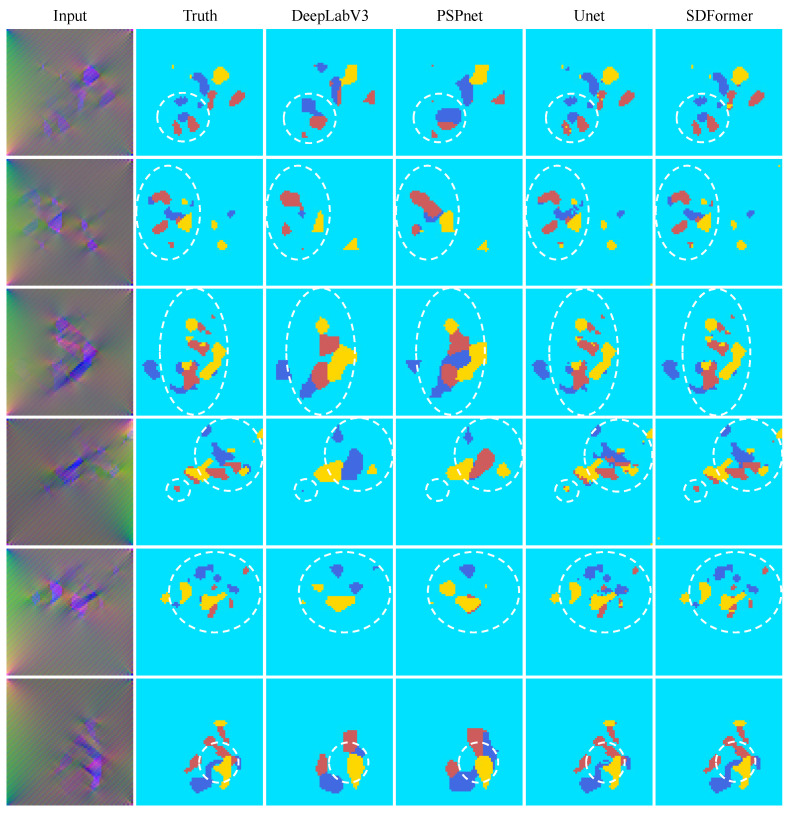
Test result of DeepLabV3, PSPnet, UNet, and SDFormer on the plate dataset.

**Figure 11:**
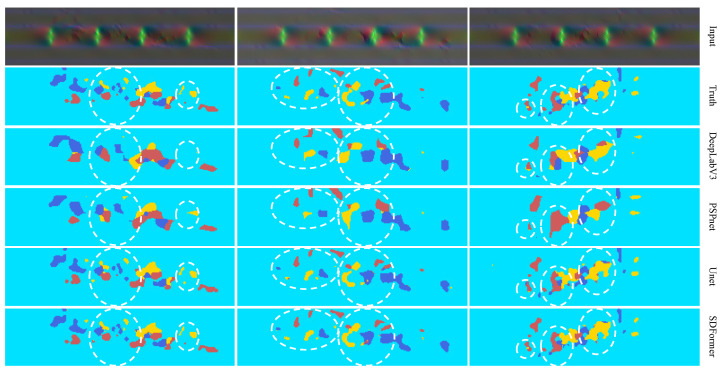
Test result of DeepLabV3, PSPnet, UNet, and SDFormer on the sleeper beam dataset.

Figure 10 compares the test results of PSPnet, DeepLabV3, UNet, and SDFormer on the plate dataset, and Figure 11 compares the test results on the sleeper beam dataset. Comparing the test results of each model on the two datasets, it could be found that for structural damage, DeepLabV3 and PSPnet could only give the approximate damage location, but could not predict the detailed parts of the damage. In contrast, UNet and SDFormer could give a prediction map very close to the real damage map. Further observing the white dashed circle in Figure 10 and Figure 11, it can be seen that SDFormer predicts the edge of the structural damage area more smoothly and accurately than UNet. This is due to the powerful self-attention module of the swin transformer block.

Table 1 and Table 2 summarize the MIoU performance, floating-point operations per second (FLOPs), and number of parameters (params) of DeepLabV3, PSPnet, UNet, and SDFormer on the plate dataset and the sleeper beam dataset. It can be seen that the MIoU performances of SDFormer and UNet far exceed those of PSPnet and DeepLabV3 in both datasets, and they were in an absolute leading position. Compared with UNet, SDFormer has higher MIoU performance and less parameters. Higher FLOPs means that the calculation requirements of SDFormer would be higher, but it is still within the acceptable range. Moreover, because the structure of the sleeper beam is more complex than that of the plate, the MIoU performance of each model was reduced on the sleeper beam dataset.

### 3.3. Anti-Noise Experiment Setup

Anti-noise performance is also a key capability for industrial applications. In order to test the anti-noise performance of SDFormer proposed in this paper, anti-noise experiments based on plate dataset and sleeper beam dataset are set up in this section. In this section, an anti-noise experiment is set up to observe the MIoU performance transformation of the model trained with noiseless data when the noise level increases. In the anti-noise experiment, the normal distribution noise was directly added to the strain field map to simulate the noise interference in the actual detection, and the model trained in Section 3.2 would be used for testing to observe the change of their MIoU performance with the increase of noise level. The noise setting formula is as follows:

(23) $$\hat{x}=\left(1+\mu \cdot N\right)x$$

where *x* is the original strain field map, $\hat{x}$ is the strain field map with noise, *N* is the normal distribution random number tensor, its size is consistent with *x*, and μ is the noise level.

In this section, five noise levels of 4%, 8%, 12%, 16%, and 20% are set for testing. The evaluation metric of anti-noise experiment was MIoU. Similarly, this section compares the anti-noise performance of four pretrained models, PSPnet, DeepLabV3, UNet, and SDFormer, on the plate dataset and the sleeper beam dataset.

### 3.4. Anti-Noise Result

**Figure 12:**
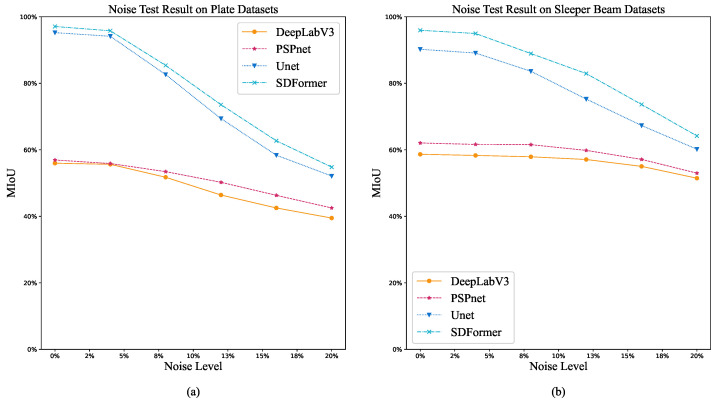
Anti-noise test result. (**a**) Anti-noise test result on the plate dataset. (**b**) Anti-noise test result on the sleeper beam dataset.

Figure 12 shows the trend of MIoU performance of DeepLabV3, PSPnet, UNet, and SDFormer models in plate dataset and sleeper beam dataset with the improvement of noise level, and Table 3 and Table 4 summarize the anti-noise performance of the proposed model and comparison of models in these two datasets. With the improvement of noise level, the MIoU performance of each model decreases gradually. Compared with the other three models, SDFormer could always maintain the best MIoU performance. This shows that SDFormer has good anti-noise ability and can maintain a good identification accuracy under certain noise conditions.

## 4. Discussion

Through the above comparison between numerical results and anti-noise experiment, it was found that the effects of SDFormer and UNet on damage segmentation are much better than DeepLabV3 and PSPnet because they both use the structure of skip connection. The skip connection structure makes the fusion of low-level features and high-level features of the model smoother. More low-level features could be effectively retained, which improves the prediction performance of the whole model. Further comparing the test results in Figure 10 and Figure 11, it could be found that the prediction result of SDFormer for the edge of the damaged area is closer to the real label than that of UNet. This is due to the self-attention module in SDFormer’s swin transformer block, which effectively enhances the weight of structural damage parts in the feature map, and then improves the overall performance of the model. With the increase of strain field map noise, SDFormer still obtains the effect better than other models, which fully shows the superiority of the model proposed in this paper. The framework of using strain field data to train a neural network to predict structural strain damage proposed in this paper is feasible, and can realize real-time damage identification.

However, it is undeniable that due to the lack of measured data in practical application scenarios, the SDFormer network proposed in this paper can only be trained and verified with the data simulated by finite element model. Labeling the strain field data from sensors is costly work, which may be a major obstacle to the promotion of the method, but it does not affect the effectiveness of this method. The problem of high data cost can be replaced by simulation data close to the real situation. In addition, although the number of parameters in SDFormer is less than UNet, its time-consumption is three times that of UNet, which will reduce the identification speed of SDFormer when it takes strain damage identification of large structures. However, due to the long time consumed by the generation of strain damage, the requirement of real-time damage identification would not be too high. Therefore, the current identification speed of SDFormer is capable of meeting the requirements.

In future work, we will explore the feasibility of applying the simulation data training model to the real structure scene, and find a method to obtain the real sensor data at a low cost.

## 5. Conclusions

In this paper, a novel U-shaped high-efficiency structural strain damage identification neural network, i.e, SDFormer, is proposed. In this method, the strain field data is converted into the form of a strain field map and input into SDFormer. The numerical simulation and experimental study were conducted on a simply supported plate and a sleeper beam of a rail train. The damage identification performance and anti-noise performance of SDFormer were studied through numerical analysis, and compared with the advanced convolutional neural network. Based on theoretical analysis, numerical experiments, and anti-noise experiments, the following conclusions can be obtained:

1. A novel structural strain damage identification strategy is proposed in this paper. This strategy takes the strain field map of the structure as the input, and uses the image segmentation algorithm to identify the damage location and level. This damage identification process is simple and there is no need for complex damage index design. On the premise of ensuring the accuracy, it can greatly simplify the process of damage identification and improve the efficiency.
2. According to the results of numerical experiments, compared with the advanced convolutional neural network, the SDFormer can achieve better damage identification performance with fewer parameters. The damage identification results of SDFormer are closer to the real damage map than those of the comparison model, which shows that SDFormer has excellent structural strain damage identification performance.
3. The results of anti-noise experiment show that SDFormer can still maintain better identification performance than that of the comparison models, although the identification performance of SDFormer decreases under the influence of different noise levels, which illustrates that the SDFormer has good noise resistance and robustness.

## Figures and Tables

**Table 1:** Result on plate dataset.

Methods	Backbone	Input Size	MIoU. (×100%)	FLOPs	Params. (M)
DeepLabV3	ResNet-50	64×64	55.97	2561 M	39.63
PSPnet	ResNet-50	64×64	56.93	161 M	1.75
UNet	ResNet-50	64×64	95.21	669 M	32.5
SDFormer(ours)	-	64×64	97.07	1807 M	18.8

**Table 2:** Result on Sleeper beam dataset.

Methods	Backbone	Input Size	MIoU. (×100%)	FLOPs	Params. (M)
DeepLabV3	ResNet-50	64×264	58.65	102.41 G	39.63
PSPnet	ResNet-50	64×264	62.05	6.51 G	1.75
UNet	ResNet-50	64×264	90.21	26.74 G	32.5
SDFormer(ours)	-	64×264	95.93	72.29 G	18.8

**Table 3:** MIoU of anti-noise test on plate dataset.

Methods	μ=0%	μ=4%	μ=8%	μ=12%	μ=16%	μ=20%
DeepLabV3	55.97	55.63	51.74	46.40	42.51	39.48
PSPnet	56.93	55.86	53.43	50.23	46.31	42.51
UNet	95.21	94.13	82.61	69.38	58.37	52.11
SDFormer(ours)	97.07	95.78	85.37	73.56	62.71	54.79

**Table 4:** MIoU of anti-noise test on sleeper beam dataset.

Methods	μ=0%	μ=4%	μ=8%	μ=12%	μ=16%	μ=20%
DeepLabV3	58.65	58.31	57.89	57.08	55.01	51.46
PSPnet	62.05	61.61	61.54	59.82	57.11	52.98
UNet	90.21	89.09	83.60	75.25	67.29	60.17
SDFormer(ours)	95.93	94.96	88.93	82.91	73.62	64.17

## Data Availability

Not applicable.
